# Kinetic modeling of nicotine in mainstream cigarette smoking

**DOI:** 10.1186/s13065-016-0206-8

**Published:** 2016-10-12

**Authors:** Joshua Kibet, Caren Kurgat, Samuel Limo, Nicholas Rono, Josephate Bosire

**Affiliations:** 1 Department of Chemistry, Egerton University, P.O Box 536, Egerton, 20115 Kenya; 2 Department of Physics, University of Eldoret, P.O Box 1125, Eldoret, 30100 Kenya

**Keywords:** Kinetic modeling, Rate of destruction, Nicotine, Puff time

## Abstract

**Background:**

The attempt to understand the kinetic behavior of nicotine in tobacco will provide a basis for unraveling its energetics in tobacco burning and the formation of free radicals considered harmful to the cigarette smoking community. To the best of our knowledge, the high temperature destruction kinetic characteristics of nicotine have not been investigated before; hence this study is necessary especially at a time addiction science and tobacco research in general is gaining intense attention.

**Methods:**

The pyrolysis of tobacco under conditions simulating cigarette smoking in the temperature region 200–700 °C has been investigated for the evolution of nicotine and pyridine from two commercial cigarettes coded ES1 and SM1 using gas chromatography hyphenated to a mass selective detector (MSD). Moreover, a kinetic model on the thermal destruction of nicotine within a temperature window of 673 and 973 K is proposed using pseudo-first order reaction kinetics. A reaction time of 2.0 s was employed in line with the average puff time in cigarette smoking. Nonetheless, various reaction times were considered for the formation kinetics of nicotine.

**Results:**

GC–MS results showed the amount of nicotine evolved decreased with increase in the puff time. This observation was remarkably consistent with UV–Vis data reported in this investigation. Generally, the temperature dependent rate constants for the destruction of nicotine were found to be $k = 2.1\; \times \;10^{6} T^{n} \; \times \;e^{{ - \,\frac{108.85}{RT}}}$ s^−1^ and $k = 3.0\; \times \;10^{7} T^{n} \; \times \;e^{{ - \,\frac{136.52}{RT}}}$ s^−1^ for ES1 and SM1 cigarettes respectively. In addition, the amount of nicotine evolved by ES1 cigarette was ~10 times more than the amount of nicotine released by SM1 cigarette.

**Conclusion:**

The suggested mechanistic model for the formation of pyridine from the thermal degradation of nicotine in tobacco has been found to be agreement with the kinetic model proposed in this investigation. Consequently, the concentration of radical intermediates of tobacco smoke such as pyridinyl radical can be determined indirectly from a set of integrated rate laws. This study has also shown that different cigarettes can yield varying amounts of nicotine and pyridine depending on the type of cigarette primarily because of potential different growing conditions and additives introduced during tobacco processing. The activation energy of nicotine articulated in this work is consistent with that reported in literature.

**Graphical abstract:**
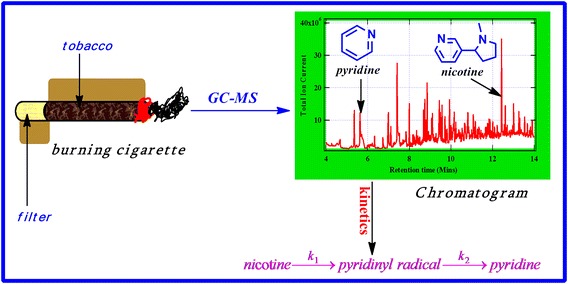
The anatomy of tobacco cigarette and the major chemistry involved during combustion (pyrolysis, GC–MS analysis, and kinetic modeling)

**Electronic supplementary material:**

The online version of this article (doi:10.1186/s13065-016-0206-8) contains supplementary material, which is available to authorized users.

## Background

Tobacco smoke is a highly dynamic and very complex matrix consisting of over 6000 compounds which makes a cigarette behave like a chemical reactor where several complex chemical processes take place during pyrolysis [1–6]. Pyrolysis can be described as the direct decomposition of an organic matrix to obtain a range of reaction products in limited oxygen [7–10]. Accordingly, the thermal degradation reaction mechanisms are complex and therefore it is necessary to simplify input parameters and physical properties in order to simulate the largest possible influence on the overall kinetic characteristics of biomass pyrolysis including tobacco [8, 9]. A kinetic scheme of biomass pyrolysis must therefore involve the solution of a high-dimensional system of differential equations [11–13].

The thermal destruction of nicotine in this investigation was conducted within a temperature window of 673 and 973 K at an average reaction time of 2.0 s as reported in literature [14–16]. For simplicity, a consecutive first order reaction with rate constants *k*_1_ and *k*_2_ has been considered in which a global kinetic model [17–20] was employed to obtain the kinetic parameters for the thermal destruction of nicotine in mainstream cigarette smoking. Accordingly, pseudo-unimolecular reactions were applied in which the empirical rate of decomposition of the initial product is first order and expressed by Eq. 1.

1 $$C = C_{o} e^{ - kt}$$

where C_o_ and C are respective concentrations of the reactant at time, t = 0, and time, t = 2.0 s, while *k* is the pseudo-unimolecular rate constant in the Arrhenius expression (cf. Eq. 2).

2 $$k = Ae^{{ - \;\frac{Ea}{RT}}}$$

A is the pre-exponential factor (s^−1^), *Ea* is the activation energy (kJmol^−1^), R is the universal gas constant (8.314 JK^−1^mol^−1^), and T is the temperature in K. Despite all the criticisms against the Arrhenius rate law, it remains the only kinetic expression that can satisfactorily account for the temperature-dependent behavior of even the most unconventional reactions including biomass pyrolysis [9]. The integrated form of the first order rate law (cf. Eq. 3) was used to calculate the rate constant for the pyrolysis behavior of tobacco at a reaction time of 2.0 s.

3 $$k = \ln \left( {\frac{Co}{C}} \right)\frac{1}{t}$$

The activation energy was determined from the Arrhenius plots (ln *k* vs. $1/T$) which establishes a linear relationship between the pre-exponential factor *A* and the rate constant *k* as given by Eq. 4, where ln *A* is the y-intercept and $- \frac{Ea}{RT}$ is the slope.

4 $$\ln k = \ln A - \frac{Ea}{RT}$$

To the best of our knowledge, there is no known destruction kinetic modeling of nicotine reported in literature. Consequently, this is perhaps the first such study on the destruction kinetics of nicotine. Although, the results obtained in this study are estimated from experimental data and may require further tests, we believe this an important step in the study of kinetics of reaction products in complex biomass materials such as plant matter. In this work, we have used GC-Area counts to determine the destruction rate constants because according to the first order reaction kinetics (Eq. 3, vide infra) the ratio of concentrations at various temperatures is a constant. Therefore, calibration of nicotine will still achieve similar results.

The primary focus of this study is to give a general kinetic account of the destruction kinetics of nicotine and demonstrate how the concentration of intermediates, in this case, pyridinyl radical can be determined indirectly and estimate the kinetic parameters of nicotine in ES1 and SM1 cigarette. The kinetics of nicotine destruction is based on high temperature regimes characteristic of cigarette burning [16, 21]. The results reported in this investigation are no doubt different from the kinetics of nicotine inhaled into the blood system which is beyond the scope of this study. Therefore, this work considers only the gas-phase kinetics of nicotine deemed fundamental towards understanding the inhalation kinetics of mainstream cigarette smoke. Furthermore, attempts have been made to identify and describe kinetically the intermediate radicals produced by the thermal degradation of nicotine from two different commercial cigarette samples (ES1 and SM1). Radicals such as pyridinyl radical which is the focus of this work have been known to cause serious health impacts because they are highly reactive towards biological tissues such as DNA, lipids, and microphages [22–25]. Free radicals such as pyridinyl radical has the ability to generate reactive oxygen species when it reacts with biological tissues and thus accelerating the growth of tumours, cancer cells, cell injury and oxidative stress [25–27].

From a quantum chemical perspective, the scission of the phenyl C–C linkage in nicotine has been explored using the density functional theory (DFT) in order to determine the energetics for the formation of pyridinyl radical from pure nicotine (in absence of other tobacco components). Although this is critical in understanding the mechanistic formation of pyridine from nicotine, it will only be discussed briefly.

## Experimental protocol

### Materials

The heater (muffle furnace) was purchased from Thermo Scientific Inc., USA while the quartz reactor was locally fabricated in our laboratory by a glass-blower. Commercial cigarettes coded SM1 and ES1 (for confidential reasons, cannot be revealed) were purchased from retail outlets and used without further treatment. Methanol (purity >>99 %) used to dissolve cigarette pyrolysate was purchased from Sigma Aldrich Inc. (USA). All experiments in this work were conducted under ISO conditions reported in Reference [16].

### Sample preparation

Processed tobacco (from ES1 and SM1) of 50 ± 0.2 mg was weight and packed in a quartz reactor of dimensions: i.e. 1 cm × 2 cm (volume ≈ 1.6 cm^3^). The tobacco sample in the quartz reactor was placed in an electrical heater furnace whose maximum heating temperature is 1000 °C. The tobacco sample was heated in flowing nitrogen (pyrolysis gas) and the smoke effluent was allowed to pass through a transfer column and collected in 10 mL methanol in a conical flask for a total pyrolysis time of 2 min and sampled into a 2 mL crimp top amber vials for GC–MS analysis. The pyrolysis gas flow rate was designed to maintain a constant residence time of 2.0 s representative of cigarette smoking [14–16, 28]. The goal of many studies, however; is to establish the relationship between tobacco constituents and smoke products under conditions that simulate actual human smoking, but this desire remains a challenge because of the large number of processes occurring inside a burning cigarette involving varying temperatures and changes in oxygen concentration [3, 4]. It turns out that the burning conditions in a cigarette change significantly from the way the cigarette burns from the oxygen rich peripheral surface towards the interior of the cigarette where oxygen is either low or generally absent [28]. This combustion experiment was conducted under conventional pyrolysis described in literature [29] and the evolution of nicotine and pyridine were monitored between 200 and 700 °C as shown in Fig. 5.

### GC–MS determination of nicotine and pyridine from ES1 and SM1 tobacco

Analysis of nicotine and pyridine was carried out using an Agilent Technologies 7890A GC system connected to an Agilent Technologies 5975C inert XL Electron Ionization/Chemical Ionization (EI/CI) with a triple axis mass selective detector, using HP-5MS 5 % phenyl methyl siloxane column (30 m × 250 µm × 0.25 µm). The temperature of the injector port was set at 200 °C to vaporize the organic components for GC–MS analysis. The carrier gas was ultra-high pure (UHP) helium (99.999 %) and the flow rate was 3.3 mL min^−1^. Temperature programming was applied at a heating rate of 15 °C for 10 min, holding for 1 min at 200 °C, followed by a heating rate of 25 °C for 4 min, and holding for 10 min at 300 °C. Electron Impact ionization energy of 70 eV was used. To ensure that the right compounds were detected, standards were run through the GC–MS system and the peak shapes as well as retention times were compared with those of nicotine and pyridine. The data was run through the NIST and the Agilent Chemstation library databases—MS-fragmentation patterns, as additional tools to confirm the identity of the compounds (nicotine and pyridine) [29]. The MS-fragmentation patterns for these compounds are presented in the support information (Additional file 1): S1(MS-Fragmentation pattern of nicotine) and S2(MS-Fragmentation pattern of pyridine). Experimental results were averaged replicates of two or more data points.

### GC–MS and UV–Vis analysis of nicotine in ES1 cigarette

**Fig. 1:**
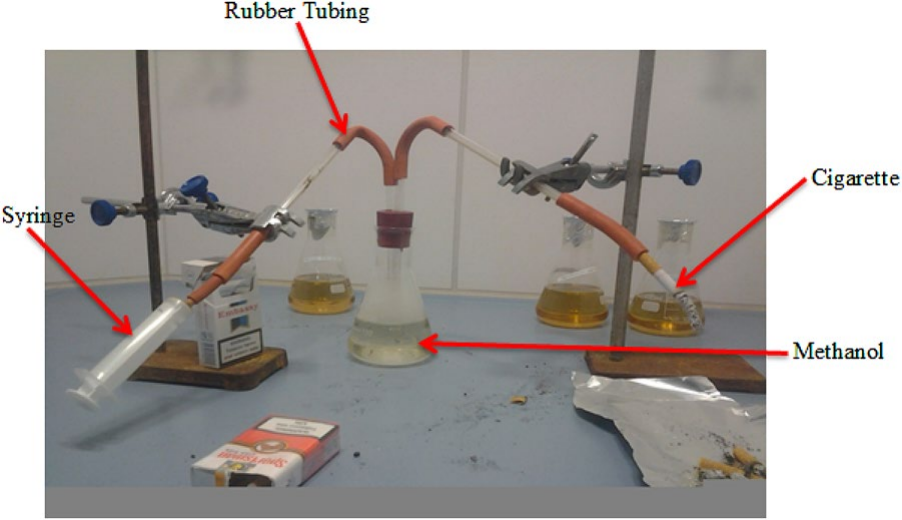
Apparatus set up for trapping cigarette smoke from cigarette burning

The rate of formation of nicotine from ES1 cigarette was determined experimentally at modest puff times (2, 5, and 10 s) using laboratory designed apparatus (Fig. 1). For every puff time, the concentration of nicotine was determined using a GC–MS hyphenated to a mass selective detector as discussed in the section above. To qualify the characteristic kinetics for the formation of nicotine at various puff times, the absorbance measurements of nicotine were taken and absorbance curves plotted. The results remarkably were similar to the GC–MS data. Maximum absorbance of nicotine in UV–Vis occurred at 220 nm. The absorbance was confirmed by running nicotine standard through the UV–Vis instrument. Methanol was used as a blank in UV–Vis analysis. The model of the instrument used for UV–Vis analysis was SHIMADZU, UV 1800.

### The kinetic model

**Fig. 2:**
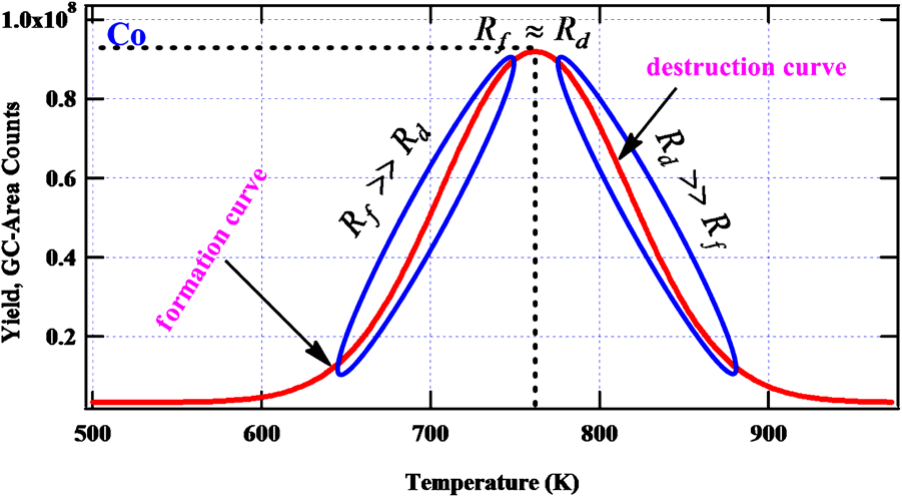
The relationship between the rates of formation of the intermediate product (R_f_) vs. the rate of destruction (R_d_). Co is taken as the maximum concentration of the reaction product

During the kinetic modeling of nicotine from the thermal degradation of tobacco biomass, decent assumptions were considered (Fig. 2): (1) the rate of formation of nicotine prevails the rate of destruction, (2) at the peak of the curve, the rates of formation and destruction are approximately the same, and (3) as the temperature is increased, the rate of destruction overwhelms the rate of formation. These assumptions are made based on the fact that pyrolysis of tobacco leads to the formation of nicotine, one of the major tobacco alkaloids as articulated in literature [6, 24, 30, 31]. This is consistent with our experiments which show that the pyrolysis of tobacco yields significant amounts of nicotine (Fig. 4). Therefore, from these assumptions, it is possible to determine the apparent kinetic parameters for the destruction of nicotine from the temperature dependence of its yields. A simple single step reaction mechanism during the thermal degradation of nicotine as presented in Eq. (5) is considered. Although tobacco pyrolysis is very complex, we believe some understanding on the kinetic behavior of certain reaction products from basic kinetic equations can be deduced. Therefore, modeling does not necessarily need to be complex to describe complex reactions systems. In essence, even simple models based on relevant assumptions may yield reasonable results as presented in this work, and compared with literature data.

5 $$Nic \mathop{\longrightarrow}\limits_{}^{k_{1}} I \mathop{\longrightarrow}\limits_{}^{k_{2}} Product$$

Conventionally, the differential rate laws for each species *Nic* (nicotine), *I* (intermediate), and the final *product* are given by Eqs. 6, 7, and 8 respectively.

6 $$\frac{d\,[Nic]}{dt} = - k_{1} [Nic]$$

7 $$\frac{d\,[I]}{dt} = k_{1} [Nic] - k_{2} [I]$$

8 $$\frac{d\,[Product]}{dt} = k_{2} [I]$$

If these equations are solved analytically, then the integrated rate laws are as given by Eqs. 9 and 10.

9 $$[Nic] = [Nic]_{0} e^{{ - k_{1} t}}$$

Equations 10 and 11 give the respective concentrations of the intermediate *I* and the *product* at any time t.

10 $$[I] = \frac{{k_{1} [Nic]_{0} }}{{k_{2} - k_{1} }}\left( {e^{{ - k_{1} t}} - e^{{ - k_{2} t}} } \right)$$

11 $$[Product] = [Nic]_{0} \left[ {1 + \frac{{k_{1} }}{{k_{1} - k_{2} }}\left( {k_{2} e^{{ - k_{1} t}} - k_{1} e^{{ - k_{2} t}} } \right)} \right]$$

In order to simplify Eq. 11 further, we will assume that step two (Eq. 5) is the rate determining step so that *k*_2_ << *k*_1_ and thus the term $e^{{ - k_{1} t}}$ decays more rapidly than the term $e^{{ - k_{2} t}}$ [32]. Therefore Eq. 11 reduces to Eq. 12. This assumption is valid based on previous studies documented in literature [11, 32, 33].

12 $$[Product] = [Nic]_{0} \left( {1 - e^{{ - k_{2} t}} } \right)$$

## Results and discussion

**Fig. 3:**
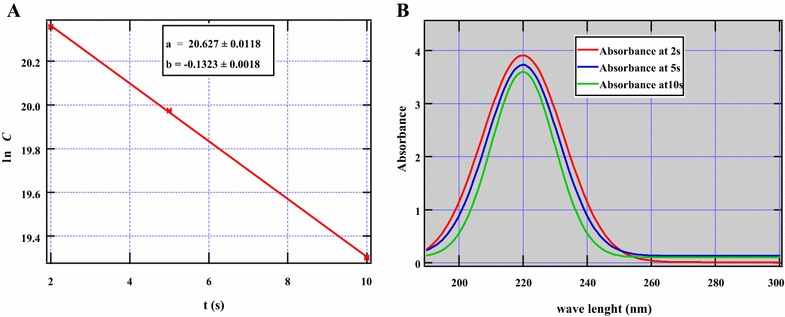
Formation kinetics of nicotine (**A**) and absorbance of nicotine at various puff times (**B**) in ES1 cigarette

To mimic actual cigarette smoking conditions, smoking apparatus were designed according to ISO 3402:1999 standards [16]. Whereas the destruction kinetics of nicotine was explored for both ES1 and SM1 cigarettes, only ES1 cigarette was investigated for nicotine formation. For formation kinetics, smoking residence times usually representative of real world cigarette smoking conditions (2, 5, and 10 s) were explored. Consequently, a plot of ln *k* as a function of puff (smoking) time yielded a straight line with a slope of −0.1323 (Fig. 3) from which the formation rate constant of nicotine (0.13 s^−1^) was calculated. The plot, although an estimation from restricted smoking times is consistent with first order reaction kinetics. The original amount of nicotine in ES1 cigarette was estimated from the y-intercept and established to be 9.1 × 10^8^ GC-Area counts. This value is remarkably close to that obtained from experimental modeling of tobacco burning from ES1, ~8.0 × 10^8^ GC-Area counts.

Interesting data have been reported in this work concerning the decrease of nicotine with smoking times (Fig. 3A, B). This suggests that longer residence times may lead to possible side reactions which result in the conversion of nicotine to other by-products. It is well known in literature that shorter residence times minimize secondary reactions but longer residence times may lead to radical formation, recombination, and pyrosynthesis of new by-products [29, 34]. Thus, these processes reduce the yield of the parent compound, in this case, nicotine. The UV–Vis data was basically qualitative but remarkably corroborates GC–MS data. Therefore, the longer the smoking times the lower the concentration of nicotine reaching the lungs of the cigarette smoker. Longer puff times may be beneficial to the smoking community based on the results obtained from this work.

### Molecular distribution of nicotine and pyridine

**Fig. 4:**
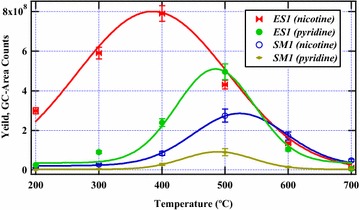
Evolution of nicotine and pyridine from ES1 and SM1 cigarette tobacco

The product distribution of nicotine in the temperature region 200–700 °C is presented in Fig. 4. Clearly, ES1 cigarette yielded high levels of nicotine and pyridine in comparison to SM1 cigarette. The nicotine levels from the two commercial cigarettes peaked at different pyrolysis temperatures. For instance, nicotine from ESI peaked at 400 °C while nicotine from SM1 peaked at about 500 °C. Interestingly, pyridine from the two cigarettes reached a maximum at about 500 °C. The two cigarettes, based on this data are significantly different. This result may be attributed to possible different growing conditions and additives during the processing of the two cigarettes. Interestingly, the total nicotine content in the entire pyrolysis range in ES1 tobacco was ~10 times the amount of nicotine released by SM1 tobacco in the same pyrolysis temperature region (200–700 °C). This may imply that SM1 cigarette is much safer than ES1 cigarette based on nicotine and pyridine data alone presented in this study. Accordingly, a close examination of the curves in Fig. 4 indicates that nicotine from the pyrolysis of tobacco is formed even at lower temperatures than the lowest temperature selected in this study (200 °C). This behaviour is explained in literature [6]. Accordingly, Forster et al. [6] proposes that the concentration of nicotine should increase with increase in the pyrolysis temperature hence the shift in nicotine yields at 200 °C as presented in Fig. 4.

**Fig. 5:**
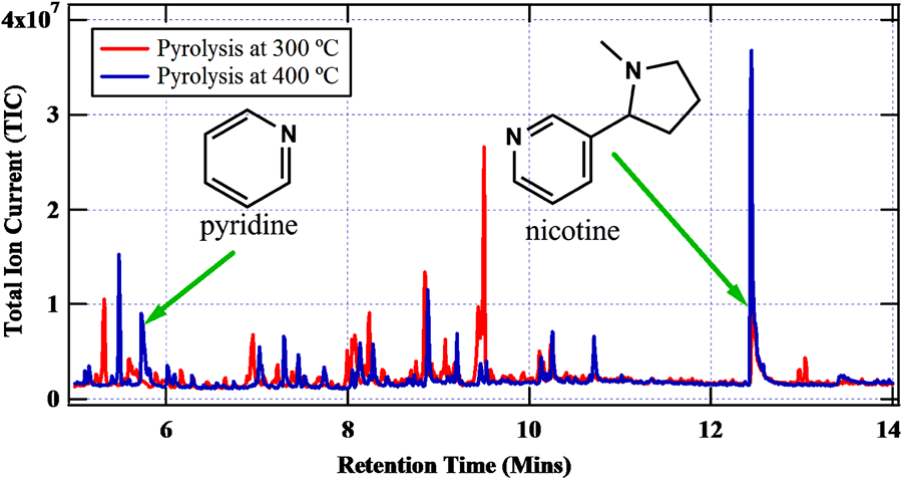
Overlay chromatograms showing the peaks for pyridine and nicotine for the pyrolysis of ES1 tobacco at 300 °C (*red line*) and 400 °C (*blue line*)

The overlay chromatograms showing the formation of nicotine and pyridine at two pyrolysis temperatures (300–400 °C) is presented in Fig. 5. Clearly, from Fig. 5, nicotine has a high intensity at 400 °C in agreement with predictions made by Forster et al. [6]. The intensity of pyridine also increases with increase in temperature. Nonetheless, like other reaction products of tobacco and other biomass pyrolysis, nicotine peaks between 300 and 500 °C before decreasing significantly with increase in temperature [29, 30, 35] (Fig. 4). The region where the concentration of nicotine begins to decrease with increase in temperature as illustrated in Fig. 2 forms the basis for modeling the destruction kinetics of nicotine which is the main subject of this investigation.

### Destruction kinetics of nicotine

**Fig. 6:**
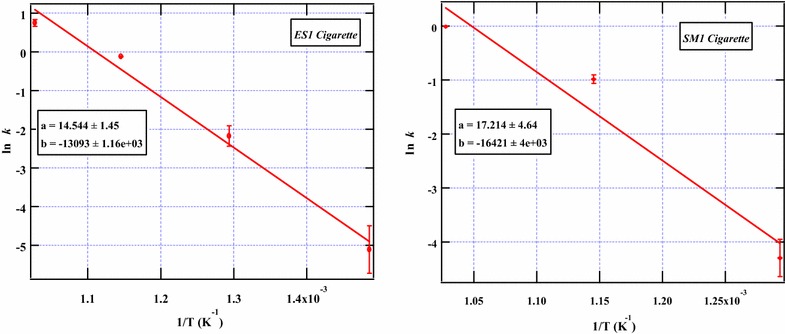
Arrhenius plots for the destruction kinetics of nicotine in ES1 and SM1 cigarette tobacco

The destruction kinetics revealed that nicotine from ES1 has activation energy of 108.85 kJmol^−1^ while SM1 has activation energy of 136.52 kJmol^−1^ (Table 1). This implies that the two cigarettes may have different matrix composition. Thus the activation energies of nicotine in the two cigarettes may not necessarily be the same considering the fact that additives of varying composition introduced during cigarettes processing may act as catalysts and ultimately reduce the activation energy of a given compound in a complex biomass material such as tobacco. Remarkably, the activation energy determined from this study is comparable to that documented in literature in which the average activation energy of nicotine was found to be 120 kJmol^−1^ [6]. Moreover, the activation energies determined from this work are similar to the results from the kinetic modeling of the pyrolysis of other biomass materials such as cellulose [11]. Arrhenius plots for the destruction of nicotine from the cigarettes under study are presented in Fig. 6. Nonetheless, in modeling the destruction kinetics of nicotine, we are aware that the kinetic characteristics of a given heterogeneous system such as plant matter may change during the process of pyrolysis and so it is possible that the complete reaction mechanism cannot be represented adequately by a specific kinetic model [9, 36]. Although we have assumed a linear relationship between ln *k* and $1/T$ we note that not all reactions will necessarily obey this relation. Therefore in order to estimate the Arrhenius dependent rate constants consistent with experimental rate constants, the modified Arrhenius rate expression is applied.

**Table 1:** The Arrhenius parameters for the destruction of nicotine from the pyrolysis of ES1 and SM1 cigarette tobacco

Cigarette type	Ea (kJmol^−1^)	A (s^−1^)
ES1	108.85	2.1 × 10^6^
SM1	136.52	3.0 × 10^7^

13 $$k = AT^{n} e^{{ - \;\frac{Ea}{RT}}}$$

For a given temperature, since the rate constant has been determine experimentally and all the other parameters are known, the value of n can be determined from Eq. 13. For instance, the value of n at 673 K was determined and found to be 0.55 and 1.05 for the destruction of nicotine in ES1 and SM1 cigarettes respectively. Equation 13 can be used to calculate the value of n at any particular temperature, since the rate constants are temperature dependent.

The destruction rate constant *k*_1_ at 673 K for ES1 was 0.31 s^−1^ while that of SM1 at the same temperature was estimated as 0.74 s^−1^. At the highest pyrolysis temperature (973 K), the respective rate constants were 2.12–1.0 s^−1^. Accordingly, the average destruction rate constant for ES1 was found to be 1.11 s^−1^. Table 1 presents the Arrhenius parameters from the destruction kinetics of nicotine (Activation energies and Arrhenius factors). Whereas the activation energies are comparably close, the pre-exponential factors for the two cigarettes under study differ by a whole magnitude.

If wish to calculate the rate constant *k*_2_ for the formation of the product, for instance pyridine (a by-product of nicotine pyrolysis), then we will need to use the differential rate law provided in Eq. 12. To be able to do this, serious assumptions have to be taken into account. For instance, one of the major by-products from the destruction of nicotine pyrolysis must be pyridine [30, 37]. This assumption is valid if we take into consideration the reactive nature of the H radical relative to the methyl radical which may yield 3-methylpyridine (a minor product) [20, 29]. Furthermore it has been proven experimentally that one of the major by-products from the thermal destruction of nicotine is pyridine [4, 30]. These findings corroborate our kinetic model on the thermal destruction of nicotine at high temperature smoking regimes.

Therefore, by substituting the original concentration of nicotine for ES1 (8.0 × 10^8^ GC-Area counts) and the maximum concentration of the product, in this case, pyridine (4.4 × 10^8^ GC-Area counts) into Eq. 12, vide supra, the value of *k*_2_ was computed and found to be 0.13 s^−1^. This shows that the value of *k*_2_ is less than the value of *k*_1_ by 1 magnitude. Secondly, since the rate constants *k*_1_ and *k*_2_ have been estimated, and the original value of nicotine is known, then the concentration of the intermediate, pyridinyl radical, can be calculated from Eq. 10. Accordingly, the concentration of pyridinyl radical was determined as 6.1 × 10^8^ GC-Area counts. Similar calculations were conducted for the kinetics of nicotine in SM1 cigarette and the value of *k*_2_ was estimated as 0.67 s^−1^ while its pyridinyl radical intermediate had a concentration of 3.31 × 10^8^ GC-Area counts. From these data, the concentration of pyridinyl radical in ES1 is ~2 times the concentration of pyridinyl radical in SM1.

Evidently, the sum of the concentrations of the intermediate and the proposed final product (pyridine) for each cigarette was greater than the original concentration of nicotine evolved by each cigarette. This is expected because in the pyrolysis of a complex matrix such as plant matter, various heterogeneous reactions occur. Thus the thermal degradation of nicotine may not be the only route for pyridine formation. This argument is acceptable if we consider experimentally that both nicotine and pyridine are evolved simultaneously during pyrolysis (Fig. 5). Nevertheless, nicotine destruction is suggested as the major route for the formation of pyridine [30, 37]. The ratio of original nicotine to the sum of concentrations of the intermediate (pyridinyl radical) and pyridine for ES1 and SM1 cigarettes were respectively 0.76 and 0.60. On the other hand, the ratio of pyridine (presumed the major by-product of nicotine destruction) to the original nicotine was determined as 0.55 and 0.52 for ES1 and SM1 respectively. These findings indicate that it might be possible that ~45 % of nicotine in ES1 and ~48 % in SM1 may have been transferred intact into the smoker. Schmeltz et al. [30] puts this figure at <41 %. This discrepancy may be attributed to a number of factors; the type of tobacco and the pyrolysis conditions. In our study, we have used an inert atmosphere to simulate cigarette smoking which implies extensive fragmentation may occur during the thermal degradation of tobacco resulting in high yields of pyridine as reported in literature [4].

### Mechanistic description for the formation of pyridine from nicotine

**Scheme 1:**
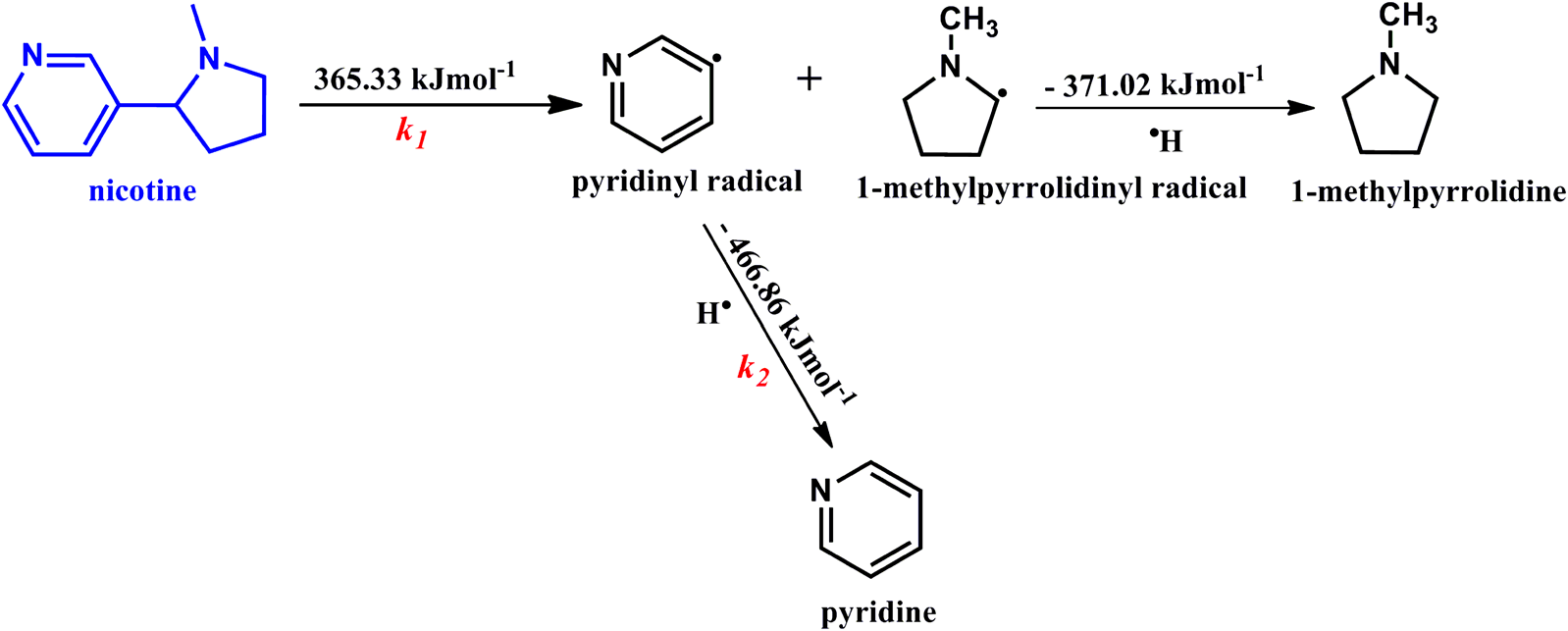
Mechanistic destruction of nicotine to radical intermediates and possible by-products

It is possible by inspection to envisage that the scission of the C–C phenyl bond in nicotine should result in the formation of pyridine despite the complex nature of pyrolytic processes taking place in plant matter such as tobacco. In order to appreciate this assumption, we have designed a mechanistic model for the formation of pyridine from nicotine as presented in Scheme 1 to support our kinetic model. Rearrangement and dehydrogenation reactions that may yield compounds such as β-nicotyrine from nicotine may not be thermodynamically feasible. This is in agreement with our experimental results in which insignificant yields of β-nicotyrine were detected. The other assumption is 1-methylpyrrolidine is a minor product. From an experimental perspective, this assumption is true because no 1-methylpyrrolidine was detected in the entire range of tobacco pyrolysis whereas significant amounts of pyridine was detected, Fig. 5, vide supra. Although, pyridine may not be the only by-product of nicotine decomposition owing to the complex processes occurring during tobacco pyrolysis, it is definitely one of the major products [6, 30]. Nonetheless, its yields depends entirely on the growing conditions of tobacco, additives introduced during tobacco processing, and the pyrolysis atmosphere in tobacco burning. This observation is clear based on the results of the two cigarettes reported in this study.

The bond dissociation energy via the rate constant *k*_1_ and the bond formation energy via rate constant *k*_2_ (scheme 1) were estimated using the density functional theory framework at the B3LYP energy functional in conjunction with 6-31G basis set. Nonetheless, the bond energies will not be discussed further because they are the subject of critical discussions in our next article. The scheme, however; proposes a plausible mechanistic pathway for the thermal degradation of nicotine to the intermediate (pyridinyl radical) and ultimately to pyridine.

### Toxicological impacts of nicotine, pyridine, and pyridinyl radical

Animal studies support biological evidence for accelerated motor activity, neurobehavioral, learning and memory deficits, and alteration of neurotransmitter function due to exposure to nicotine [38, 39]. Nicotine also affects the cardiovascular system in many ways that is by activating the sympathetic nervous system; nicotine induces increased heart rate and myocardial contraction, vasoconstriction in the skin and adrenal, reproductive problems and neural release of catecholamine [40–42]. Nicotine can also affect lipid metabolism [43], accelerate the development of atherosclerosis [44], induce endothelial dysfunction [45], and has been suspected as a carcinogen [42]. After a puff, high levels of nicotine reach the brain in 10–20 s, faster than with intravenous administration, producing rapid behavioural reinforcement [46]. On the other hand, pyridine has been implicated in the inhibition of the growth of chick chorioallantoic membrane and reproductive health issues [37, 47, 48]. In this study, the radicals including pyridinyl and 1-methylpyrrolidinyl radicals are good candidates for cell injury and oxidative stress during cigarette smoking. The molecular structure of nicotine and other alkaloid related compounds investigated in this work may covalently bond to the DNA, lipids, nuclei acids, and body cells before metabolizing into harmful by-products that are potential risks to the human health [23, 26, 27, 42]. In addition, pyridinyl radical can react with biological molecules to enhance the production of reactive oxygen species which can cause oxidative stress, tumourogens, and cancer [23, 49–52].

## Conclusion

The temperature dependent destruction kinetics of nicotine has been presented for the first time in this investigation. A mechanistic model showing the formation of pyridine from the thermal destruction of nicotine has been proposed and found to be in agreement with the kinetic model reported in this study. We therefore believe the results presented in this investigation will form the basis of further research towards understanding the fate of nicotine during cigarette smoking. The two cigarettes investigated in this work coded ES1 and SM1 have exhibited various kinetic characteristics possibly because of their different biomass composition attributed mainly to their growing conditions and additives during tobacco processing. Moreover, this study has established that the activation energy of nicotine is remarkably consistent with that reported in literature. The concentration of the intermediate (pyridinyl radical) has been estimated from kinetic modeling of nicotine. This is remarkable since the concentrations of intermediates in complex reaction systems such as biomass are usually tedious to determine experimentally.

## Additional file

10.1186/s13065-016-0206-8 This has beeb corrected accordingly under the section GC-MS determination of nicotine and pyridine in ES1 and SM1 tobacco.
