# Supplementary material for: Kinetic modeling of nicotine in mainstream cigarette smoking
Source: Chem Cent J. 2016 Oct 12;10:60. doi: 10.1186/s13065-016-0206-8 (PMC5062895; doi:10.1186/s13065-016-0206-8)
Supplement: Supplementary file 1 — 10.1186/s13065-016-0206-8 This has beeb corrected accordingly under the section GC-MS determination of nicotine and pyridine in ES1 and SM1 tobacco. [file 13065_2016_206_MOESM1_ESM.docx]

**Kinetic Modeling of Nicotine in Mainstream Cigarette Smoking**

^1^Joshua Kibet*, ^1^Caren Kurgat, ^2^Samuel Limo, ^1^Nicholas Rono, and ^1^Josephate Bosire

^1^ Department of Chemistry, Egerton University, P.O Box 536 - 20115, Egerton, Kenya

^2^Department of Physics, University of Eldoret, P.O Box 1125 - 30100, Eldoret, Kenya

**SUPPORTING INFORMATION**

The supporting information herein is the Mass spectra (MS) fragmentation patterns for nicotine pyridine

**S1:** MS-Fragmentation pattern of nicotine

**

**

**S2:** MS-Fragmentation pattern of pyridine

**

**
